# General intermediate care units: can they effectively support intensive care units and ensure patient safety?

**DOI:** 10.62675/2965-2774.20250325

**Published:** 2025-05-04

**Authors:** Thais Dias Midega, Rogério da Hora Passos, Stephan Mathias Jakob, Thiago Domingos Corrêa

**Affiliations:** 1 Hospital Israelita Albert Einstein Department of Critical Care Medicine São Paulo SP Brazil Department of Critical Care Medicine, Hospital Israelita Albert Einstein - São Paulo (SP), Brazil.; 2 University of Bern Bern Switzerland University of Bern - Bern, Switzerland.

## INTRODUCTION

Intermediate care units (IMCUs), also known as Step-Down Units (SDUs), were developed to bridge the care gap between a hospital's general wards and the intensive care unit (ICU) by offering a level of care that is more intensive than that of general wards but less resource intensive than that of full ICUs.^(1)^ Since their introduction in the early 1970s, IMCUs have evolved in terms of their physical structure and organizational aspects, such as staffing models, practices and types (general *versus* specialty-specific IMCUs), to meet the constantly changing demands of patients and health care systems.^(2)^ Patients may be admitted to the IMCU either as a step-down from the ICU after a critical illness or as a step-up from the general wards when they are deteriorating or recovering after a low-risk surgical procedure.^(2)^

Discharge from the ICU is a complex hospital process.^(3)^ Approximately 1 in 10 ICU patients discharged to the wards will be readmitted to the ICU within 48 hours,^(4)^ which is associated with increased length of hospital stay and higher hospital mortality and costs.^(5)^ Intermediate care units may provide a safe environment for patients discharged from the ICU with a given risk of clinical deterioration because they are staffed with specialized interdisciplinary care teams and can provide close monitoring.^(6)^ In the absence of an IMCU, such patients would either be transferred directly to regular wards, increasing their risk of ICU readmission and poor outcomes, or they would remain in the ICU, extending their length of stay and placing additional strain on ICU resources. This situation could preclude the ICU admission of critically ill patients with acute organ dysfunction or failure.^(6)^

A study conducted across 167 European centers revealed that in hospitals with an IMCU, there was reduced hospital mortality among ICU patients.^(7)^ The authors suggested that the monitoring and treatment provided at the IMCU for patients before ICU admission and especially after ICU discharge, could have contributed to the decreased mortality rate.^(7)^ In contrast, patients in hospitals without an IMCU likely remain in general wards longer while deteriorating, which may negatively affect outcomes.^(7)^ Additionally, the presence of an IMCU may have allowed some patients to be treated there instead of being admitted to the ICU, thereby reducing the workload on ICU staff associated with patient turnover (admissions, transfers, and discharges).^(7)^ A high ICU staff workload is associated with increased mortality rates.^(8)^

Moreover, it has been demonstrated that IMCUs can improve ICU efficiency and adequate patient allocation in hospitals.^(9–11)^ A study conducted in 78 ICUs in Brazil demonstrated that the most efficient ICUs, evaluated on the basis of standardized mortality rates using the simplified acute physiology score (SAPS) 3 and standardized resource use, were located in hospitals with IMCUs.^(9)^ Similarly, a study involving 50 ICUs in Germany revealed that hospitals with high IMCU utilization admitted younger patients with higher severity of illness to the ICU, suggesting more appropriate patient allocation.^(10)^ Furthermore, it has been demonstrated that IMCUs facilitate ICU discharge on weekends, when fewer nurses and doctors are available in general wards.^(11)^

In addition to their operational benefits, IMCUs can improve patient comfort and experience, as the environmental conditions in these units are typically more comfortable than those in ICUs.^(12)^ For example, proper management of alarms and the promotion of a quiet environment help reduce patient discomfort and stress.^(12)^ Additionally, the presence of family members in IMCUs provides continuous support and psychological benefits, positively influencing patient recovery.^(12)^

## CHALLENGES IN IMPLEMENTING INTERMEDIATE CARE UNITS

The implementation of an IMCU presents several challenges. First, to ensure high-quality care, IMCUs require sufficiently well-staffed interdisciplinary care teams that must receive continuous training.^(12)^ Second, effective monitoring is crucial for the early detection of clinical deterioration and timely interventions.^(12)^ Third, the proper selection of patients is essential, as inappropriate IMCU admissions can result in either waste of resources or adverse events and increased mortality.^(13)^ Fourth, defining appropriate IMCU manpower poses a challenge, as few studies have evaluated the appropriate staff-to-patient ratio, which can vary according to the type of IMCU.^(12)^ Finally, the economic impact of implementing an IMCU is substantial and varies on the basis of patient severity of illness, unit size, and staffing pattern.^(14,15)^

One study reported that ICU costs per patient increased after the introduction of an IMCU.^(14)^ This increase in cost was attributed to the admission of more severely ill patients to the ICU following the establishment of an IMCU.^(14)^ On the other hand, another study reported that the implementation of IMCUs reduced total hospital health care costs, particularly when most patients admitted to the IMCU were of high acuity.^(15)^ High-acuity patients were defined as patients with a therapeutic intervention scoring system-28 score of 18 points or more, indicating those who required more than 3 hours of direct patient-related nursing workload per shift.^(15)^ Therefore, the optimal criteria for selecting patients for IMCU admission, along with organizational factors such as the staff-to-patient ratio, monitoring systems, and unit structure and size, remain subjects of ongoing debates regarding their potential to positively affect the cost-effectiveness of IMCUs.

## FUTURE PERSPECTIVES AND RESEARCH

**Figure 1:**
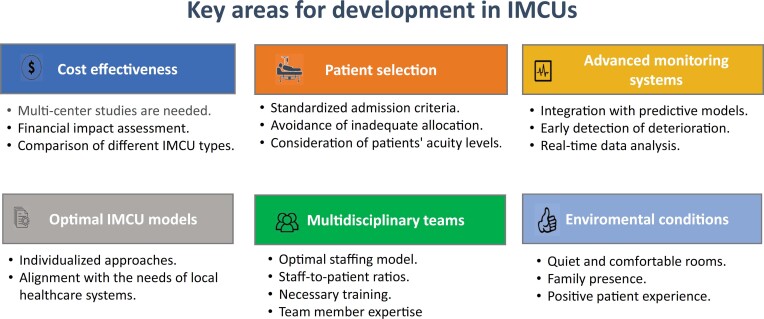
Research areas and opportunities for enhancing intermediate care unit effectiveness in supporting intensive care units and ensuring patient safety.

Future research has the potential to improve the efficiency of IMCUs (Figure 1). Key areas for research and development include establishing evidence-based guidelines for "step-up" and "step-down" admissions. These guidelines can optimize patient outcomes and ensure that IMCUs deliver appropriate levels of care.^(12)^ Additionally, studies are needed to evaluate organizational factors within IMCUs, such as the optimal unit size, staff-to-patient ratios, levels of monitoring, and staffing training. Although they may vary according to patient mix and hospital profile, sufficiently powered multicenter studies can offer a comprehensive overview of how these variables might impact patient safety and cost effectiveness. Furthermore, research into predictive analytics and machine learning holds promise for advancing patient monitoring and delivering more precise, individualized care. Integrating advanced monitoring systems, including real-time data analytics and predictive tools, could enhance the optimal IMCU capacity and efficiency.

IMCU - intermediate care unit.

## AUTHOR’S PERSPECTIVE

Intermediate care units can be effective and efficient in supporting ICUs by increasing care flexibility and improving resource allocation on the basis of patient severity. Studies on organizational factors, advanced monitoring and patient admission criteria are essential and could provide guidelines for institutions looking to implement or optimize IMCUs. However, a standardized approach may not always be feasible, as the appropriateness of any specific IMCU model relies on the case mix, institutional resources, and the facility's physical infrastructure. When appropriately adapted to local conditions and integrated into existing health care systems, IMCUs can play a critical role in enhancing patient safety and hospital efficiency.
